# Student Knowledge of University Confidential Resources and Title IX Training Effectiveness

**DOI:** 10.1177/10778012231225234

**Published:** 2024-01-09

**Authors:** Stephanie C. Kathan, Jianchao Lai, Sara Wilf, Marisa Imbroane

**Affiliations:** 1 8783 University of California, Los Angeles, Los Angeles, CA, USA; 2 Case Western Reserve University School of Medicine, Cleveland, OH, USA

**Keywords:** Title IX, sexual violence, university resources

## Abstract

Though many universities offer resources to student survivors of sexual violence, student survivors who do not know about these resources cannot utilize their services. Students who are unaware of the confidentiality status of these services may seek assistance from an on-campus service without realizing the potential consequences. Under the theory of institutional betrayal, knowledge of confidential resources may prevent experiences of institutional betrayal for students who have experienced sexual violence. This research examines predictors of student knowledge regarding the confidentiality status of university resources for student survivors. Several variables were found to be associated with student knowledge of confidential on-campus resources. Implications for Title IX training and student resource availability are discussed, including the need for additional support for specific student populations.

## Introduction

Sexual violence and sexual harassment (SVSH) are a major concern across higher education within the United States. Although many resources are available for student survivors of SVSH within higher education, students who experience SVSH can only access those resources if they have knowledge of or seek out such services. Additionally, confusion regarding which on-campus resources are confidential or not confidential may lead students who do not wish to report their experience when seeking healing services to unintentionally request assistance from on-campus resources that are required to report an incident. This study examines the knowledge of confidential and nonconfidential resources for student survivors of SVSH across the University of California system.

## Literature Review

Since 1972, Title IX of the Education Amendments has prohibited discrimination based on sex in higher education (20 U.S. Code §—Sex, 1972). The first instance of a sexual harassment case brought under Title IX was that of Alexander v. Yale, during which five women argued that Yale was violating Title IX by refusing to consider their complaints of sexual harassment by male faculty and administrators (Alexander v. Yale University, 1980). Although SVSH has been covered by Title IX since the 1980 case, SVSH continues to be a major concern for higher education students with somewhere between 37% and 56% of students experiencing unwanted sexual harassment (Klein et al., 2021). This issue disproportionality impacts women and LGBTQ + students, while students of color often face more severe consequences after an experience of SVSH (Eisenberg et al., 2021; Klein & Martin, 2021). Although scholarship on SVSH within institutions of higher education has substantially increased over the past few decades, most literature continues to focus on cis-gender heterosexual white women (Harris & Linder, 2017; Linder et al., 2020). Additionally, research studies often neglect to analyze student survivors’ experiences by social identity, such as race and gender (Harris & Linder, 2017; Linder et al., 2020).

Understanding available resources on their college campus, as well as the purpose of those resources, before an experience of SVSH can assist students in determining which resource(s) would be most beneficial in their personal healing or justice journey. In particular, students’ knowledge of the confidentiality of key resources is a notable aspect of their experience. Confidentiality may make certain resources more desirable for student survivors to seek care following an experience of SVSH (Newins & White, 2018). Under federal law, University of California (UC) schools are supposed to require all members of the UC community to attend Title IX training sessions. However, it is unclear if students recall resources for SVSH within the Title IX training. This paper utilizes a survey across UC campuses to examine predictors of student knowledge of on-campus resource confidentiality.

### Title IX Training

Almost all universities provide Title IX training to their students, faculty, and staff to ensure awareness about Title IX policies and procedures (Wiersma-Mosley & DiLoreto, 2018). Some universities, such as the UCs, outsource their Title IX training to third-party companies while others provide training developed in-house. Despite millions of people taking Title IX training every year, there is scant scholarship evaluating their efficacy, although some qualitative work has pointed to student's dissatisfaction with the Title IX training (Scwartz, 2015) and lack of awareness of basic aspects of the Title IX office's roles and responsibilities (Ruiz, 2022). Additional scholarship examines students' general awareness of campus-based resources for reporting (e.g., Schulze & Perkins, 2017), but this literature is not reviewed here as it does not evaluate Title IX training's efficacy or students' awareness of confidential resources.

As Wareham et al. (2022) note, there are few studies assessing how well students understand Title IX training and even fewer evaluating the effectiveness of the training. Two studies asked students to self-report their knowledge of mandatory reporting policies without evaluating whether students’ knowledge was correct (Newsins et al., 2018; Newins & White, 2018). One study examined whether University faculty and staff (not students) correctly knew about their university's mandatory reporting policy (Koon-Magnin & Mancini, 2023). To our knowledge, there is only one other study that evaluated University students’ correct knowledge of mandatory reporting requirements and procedures (Wareham et al., 2022). Wareham et al. (2022) utilized data from the Campus Climate Survey Validation Study across three university campuses, including the University of California, to examine whether students had knowledge of Title IX and which employees were mandatory reporters. In this study, students who received Title IX training had significantly higher levels of knowledge about what Title IX does; however, there was no significant association between receiving Title IX training and knowledge about the mandatory reporting policy (Wareham et al., 2022).

Recent research explores the connections between mandatory sexual misconduct training and student likelihood of reporting an experience of sexual assault (Htun et al., 2022). Using a quasiexperimental research design, Htun et al. (2022) examine the immediate effects of the training as they relate to Obama-era Title IX goals and guidelines and student views regarding sexism and gender. This study found that students were significantly more likely to identify cases of sexual misconduct after participating in the training compared to before the training; however, there was also an increase in misidentifying ambiguous behavior as sexual misconduct after the training (Htun et al., 2022). Interestingly, this research found that fewer students who are women stated that they would report being sexually assaulted to campus authorities after receiving the sexual misconduct training than before receiving the training (Htun et al., 2022). The authors posit that this change is related to the concretizing of the meaning of assault for survivors of potential survivors, potentially creating greater resistance to reporting among women due to the difference between men and women's concern regarding the consequences of reporting assault (Htun et al., 2022).

### Confidentiality of Resources for Student Survivors

Confidential resources are University offices and employees who are not mandated to report a student's experience of SVSH to Title IX if the student chooses to disclose the experience to them. Employees who are mandated to report are called responsible employees. There are mixed findings on students’ knowledge of confidential resources. Some research has found that overall, high rates of students know that University faculty and staff are responsible employees (Newins & White, 2018). Yet research with student survivors found they lacked awareness of their school's mandatory reporting policies (Holland et al., 2021a). Additionally, communication regarding confidential and nonconfidential resources on university websites has been found to be confusing and inaccurate or lacks information about mandatory reporting policies, making it difficult for student survivors to understand the requirements of various university services (Eno et al., 2023). Moreover, mandatory reporting policies may not support survivors’ agency and healing. In interviews with student survivors of SVSH, Holland et al. (2021a) found that survivors believed mandatory reporting policies would have more harm than benefits, such as discouraging them from seeking help from people they trusted on campus. Indeed, substantial research finds that student survivors are *less* likely to report an experience of SVSH to employees when there are mandatory reporting policies (Holland et al., 2018).

In the 10 schools comprising the UC system, there are three confidential resources: Campus Assault Resources and Education (CARE), Counseling and Psychiatric Services (CAPS), and the Office of Ombuds Services. Brief descriptions of these confidential resources are available in the methods section. Nonconfidential resources that student survivors may turn to include the Title IX office, professors, and sports coaches. Generally, the LGBT Centers on each UC campus are not confidential, although the LGBT Center at UC Davis is confidential.

### Variations in Knowledge of Campus Resources Between Student Groups

Different groups of students, for example, students of color and white students (Harris et al., 2021) or LGBQIA+ and heterosexual students (Holland et al., 2021b) have distinct experiences navigating campus resources. Therefore, determining if knowledge of campus resources differs between various student populations is essential in framing future advocacy efforts. Overall, research finds that low percentages of students can name even one campus-based resource for survivors. For example, one study of undergraduate and graduate students found that 38% could not name a single campus-based resource (Schulze & Perkins, 2017). Among undergraduate students interviewed for a multicampus study, approximately half were aware of the on-campus resources available to them (Bloom et al., 2022). In one qualitative dissertation, one-third of undergraduate students interviewed had never heard of Title IX (Schwartz, 2015). Graduate students were found in a single-campus study to have less awareness of available on-campus resources compared to undergraduate students (Whitmore, 1983). Another study suggests that graduate students have a basic understanding that on-campus resources for sexual violence exist, but that many participants in the study were unsure of where to start if they needed help (Bloom et al., 2021).

Cis-gender female students were found to be more likely to know where to go to get information regarding resources on campus (Banyard et al., 2007), but there have been mixed findings as to whether knowledge of services differs between females and males (Franklin & Menaker, 2014; Hayes-Smith & Levett, 2010; Nasta et al., 2005; Walsh et al., 2010). A qualitative study with survivors found few differences in knowledge of the Title IX office and procedures across gender and sexual identities, although of the sample only cis-gender women had reported their experience to Title IX (Holland et al., 2021a).

Although scholarship finds substantial differences in the experience of student survivors based on racial and ethnic identity (e.g., Harris et al., 2021), there is scant scholarship exploring differences in knowledge of campus resources by racial and ethnic group. One study found that knowledge of on-campus resources was lower in students of color compared to their white counterparts (Schulze & Perkins, 2017). Specifically, Tredinnick (2022) found that Hispanic student-athletes had lower awareness of policies for sexual assault reporting than student-athletes from other racial and ethnic groups. A 2016 single-campus study of Health Science major students found that although most students were able to identify on-campus resources, only students who were members of Residence Life knew what services were available at each resource (Pappalardo, 2016). Students with an increased number of exposures to messaging about sexual violence were found to have increased knowledge of campus resources (McMahon & Stepleton, 2018).

### Institutional Betrayal

Institutional betrayal occurs when harm is caused to an individual by an institution that the individual depends on or trusts, and research shows that it worsens levels of anxiety, trauma-specific sexual symptoms, dissociation, and problematic sexual functioning (Smith & Freyd, 2013, 2014). Moreover, lesbian, gay, and bisexual university students who are survivors of sexual violence are at a heightened risk of experiencing institutional betrayal (Smith et al., 2016). Variations of institutional betrayal include harm by omission of protective actions or by active commission, and harm may occur in ways that appear to be either systematic or isolated to the individual (Smith & Freyd, 2014). When the focus of betrayal is moved away from that of an individual and toward that of an institution, it becomes possible to identify patterns and areas of institutional betrayal across a system (Smith & Freyd, 2014). Measurements of institutional betrayal include failure to prevent abuse, the normalization of abusive contexts, difficult reporting procedures and inadequate responses, supporting cover-ups and misinformation, and punishing victims and whistleblowers (Smith & Freyd, 2014). Meanwhile, experiences of institutional betrayal can be prevented through transparency and the protection of institution members on the part of the institution (Smith & Freyd, 2014). The prevention of experiences of institutional betrayal may prevent experiences of trauma, posttraumatic stress disorder, and harmful experiences at a systematic level.

Sexual violence within institutions of higher education has been a central example of institutional betrayal since the theory was introduced to the psychology literature (Smith & Freyd, 2014). Within this area, institutional betrayal can be prevented through a combination of policies and procedures designed to support survivors of sexual violence, the ability for students within higher education to access resources that support their decisions if they have experienced sexual violence, and protection and prevention of sexual violence across university campuses. By examining student knowledge of confidential and nonconfidential resources across institutions of higher education, steps may be taken to improve this knowledge and potentially prevent experiences of institutional betrayal for future survivors of sexual violence.

## Methods

### Survey

The survey used within this analysis was developed by the student-led collective organization, Survivors and Allies (S + A), which advocates for survivors of sexual violence across the UC system. S + A members collaborated to develop a survey examining student awareness, utilization, and evaluation of both on- and off-campus resources available for survivors in 2020. This 20-min online survey was distributed from May to November 2021 to organizations and departments across all 10 of the UC campuses. The survey was primarily quantitative; however, several open-ended questions were included. Survey respondents were entered into a raffle for $25 gift cards as compensation for participation. The research project was approved by the University of California, Los Angeles Institutional Review Board, IRB #21-000300.

Data from the survey includes student awareness of campus-based resources for SVSH, Title IX training, and survivors’ evaluation of campus-based resources, including Title IX and UCPD. The novel survey asked students about both on-campus and off-campus resources and people they utilized for healing, allowing for a more holistic and survivor-centered analysis.

### Confidential Campus Resources

Three confidential campus resources are available to student survivors across the UC system: CARE, Counseling and Psychiatric Services (CAPS), and the Office of Ombuds Services.

#### Campus assault resources and education (CARE)

The CARE program began as the Rape Prevention Education Program (founded in 1976), as a joint program between UCPD and the Women's Center (History of CARE, n.d.). This program was renamed to CARE in 2013 for increased accessibility for survivors of additional forms of interpersonal violence in addition to rape (History of CARE, n.d.). In 2014, a University of California task force was formed to ensure CARE resources would be available to students across all 10 UC campuses after a change to UC policy ensuring compliance with the Violence Against Women Act (UC Office of the President, 2014).

The CARE program provides in-person and virtual support to UC student survivors. Although each individual CARE office is independently run, each office provides similar services for the UC student body of the individual campus. These services include healing-focused events, appointments with CARE advocates to support student survivors with their chosen next steps, and educational workshops focused on a variety of topics for the UC student population.

#### Counseling and psychiatric services (CAPS)

The CAPS program provides mental health assistance to UC students across the system's 10 campuses. Similar to the CARE program, CAPS offices are independent from one another; however, they provide similar services to each individual UC campus. These resources include in-person and telehealth services for students residing in the state of California (Services, n.d.). CAPS offers students a set number of individual therapy sessions with a mental health professional, referral services to long-term mental health care, and group sessions to support common mental health concerns for students (Services, n.d.). As CAPS provides counseling and psychological services to any student within the UC system, the focus of these services is not specifically related to sexual violence.

#### Office of ombuds services

The Office of Ombuds Services provides informal conflict resolution services to members of the UC community, acting as a neutral, independent, and confidential service (UCLA Office of Ombuds Services, n.d.). Each university campus houses its own Office of Ombuds Services, and appointments are available both in-person and virtually. Unlike CARE and CAPS, the Office of Ombuds Services does not provide counseling or psychological support to UC community members. However, it is intended to serve not only students, but also faculty, staff, and administrators across the various UCs (UCLA Office of Ombuds Services, n.d.). The Office of Ombuds Services primarily acts as a mediator for disputes or conflicts across campus; however, the office may also provide information to individuals who seek support, such as the availability of other campus resources.

### Sample

Due to the limited amount of data on genderqueer respondents, students whose identities are outside the gender binary (genderqueer/NB/agender/intersex/other, prefer not to disclose/questioning) were dropped from the analysis (*N* = 169). People who stated they were transmen or transwomen were placed into the group which accurately represents their gender (i.e., transmen were grouped with men, and transwomen were grouped with women). As this study is focused on student knowledge, faculty and staff (*N* = 106) were removed from the dataset. Additionally, listwise deletion was used across variables included within the regression analyses, meaning the analysis subsample was limited to respondents who answered each question utilized in the analyses. Social identities of participants and the total survey respondents, including race/ethnicity, UC campus, sexuality, gender, transfer student status, international student status, and graduate student status can be seen in Table 1.

**Table 1.** Social Identities of Participants and Total Survey Respondents.

	Count (percent) of analysis subsample (*N* = 677)	Count (percent) of total survey respondents (*N* = 1,384)^a^
Race/ethnicity		
Asian	186 (27.47%)	378 (27.92%)
Native American/Alaskan Native	9 (1.33%)	40 (2.95%)
Black/African American	20 (2.95%)	47 (3.47%)
Hispanic/Latino	123 (18.17%)	241 (17.8%)
Middle Eastern/North African	13 (1.92%)	25 (1.85%)
White	231 (34.12%)	432 (31.91%)
Other	5 (0.74%)	12 (0.89%)
Multiracial	89 (13.15%)	179 (13.22%)
University of California Campus		
Berkeley	90 (13.29%)	168 (13.14%)
Davis	79 (11.67%)	120 (9.38%)
Hastings	92 (13.59%)	207 (16.18%)
Irvine	128 (18.91%)	203 (15.87%)
Los Angeles	57 (8.42%)	127 (9.93%)
Merced	49 (7.24%)	88 (6.88%)
Riverside	35 (5.17%)	101 (7.9%)
San Diego	6 (0.89%)	10 (0.78%)
San Francisco	49 (7.24%)	86 (6.72%)
Santa Barbara	89 (13.15%)	160 (12.51%)
Santa Cruz	3 (0.44%)	9 (0.7%)
LGBQIA	399 (58.94%)	769 (56.92%)
Women	498 (73.56%)	852 (74.48%)
Nontransfer students	580 (85.67%)	977 (83.86%)
Domestic students	589 (87%)	1,000 (82.78%)
Graduate students	358 (52.88%)	604 (48.75%)

a Frequencies may not add up to total; respondents were not required to answer all questions.

### Measures

Various survey responses from the S + A survey were utilized within the analysis. In addition to respondent's social identities, these include knowledge of confidentiality of on-campus resources and respondent's perceived effectiveness of the Title IX training in teaching about on-campus resources.

#### Knowledge of confidentiality of on-campus resources

Within the S + A survey, students were asked to identify which on-campus resources were confidential from a list of nine resource options. This question was phrased in the following way: “Which of the following offices and people are confidential (i.e., they won’t report if you tell them that you have experienced sexual violence or assault)? Please select all that apply.” The nine offices or people who were listed were: the Title IX Office (or HSTAT at UC Davis where you would make a formal report), CARE, CAPS, the Office of Ombuds Services, professors, teaching assistants, sports coaches, UC administrative staff, and UC Student Health Clinics.

Combining the correct responses to these options, the research team developed a scale from zero to nine regarding students’ knowledge of confidentiality of on-campus resources. Table 2 displays the confidentiality status of each resource, the number of respondents who answered correctly who were used within the analysis, and the number of respondents who answered correctly across all survey respondents. Knowledge of on-campus resources was used as the dependent variable in the final analysis Table 2.

**Table 2.** Knowledge of Confidentiality of On-Campus Resources Across Respondents.

Resources	Confidential for survivors of SVSH	Count (percent) of analysis subsample correct (*N* = 677)^a^	Count (percent) of total survey respondents correct (*N* = 1,384)^a^
Title IX office	Nonconfidential	372 (53.95%)	873 (63.08%)
CARE^b^	Confidential	451 (66.62%)	751 (54.26%)
CAPS^c^	Confidential	438 (64.7%)	727 (52.53%)
Office of Ombuds Services	Confidential	164 (24.22%)	322 (23.27%)
Professors	Nonconfidential	626 (92.47%)	1,286 (92.92%)
Teaching assistants	Nonconfidential	635 (93.8%)	1,307 (94.44%)
Sports coaches	Nonconfidential	648 (95.72%)	1,321 (95.45%)
UC administrative staff	Nonconfidential	624 (92.17%)	1,287 (92.99%)
UC Student Health Clinics	Nonconfidential	441 (65.14%)	988 (71.39%)

a Frequencies do not add up to total; respondents answered yes or no to each question.

b The CARE program is the Campus Assault Resources & Education program offered through UC campuses.

c CAPS is the Counseling and Psychological Services offered through UC campuses.

#### Perceived effectiveness of title IX training

Within the S + A survey, students were asked to describe how they felt about the Title IX training's effectiveness at teaching them about which on-campus resources were confidential. This question was phrased “How effective was the Title IX training at teaching you about which on-campus resources are confidential for discussing experiences of sexual violence and sexual harassment?” Response options included “Very effective,” “Somewhat effective,” “Neutral,” “Not very effective,” and “Not at all effective.” Table 3 displays the frequencies of these responses across the subsample used for the analysis and for the respondents overall.

**Table 3.** Perceived Effectiveness of the Title IX Training Regarding Confidential Resources.

Response	Count (percent) of analysis subsample (*N* = 677)	Count (percent) of total survey respondents (*N* = 1,384)
Very effective	82 (12.11%)	137 (9.9%)
Somewhat effective	298 (44.02%)	437 (31.58%)
Neutral	152 (22.45%)	237 (17.12%)
Not very effective	109 (16.1%)	147 (10.62%)
Not at all effective	36 (5.32%)	46 (3.32%)
Missing	0	380 (27.46%)

### Analysis

We used the S + A survey dataset to examine the perceived effectiveness of Title IX training compared to student knowledge regarding UC services and their confidentiality status (confidential vs nonconfidential resources). Bivariate analysis was performed to identify the potential key covariates. Multiple regression predicting knowledge of confidentiality of on-campus resources were completed in Stata, and listwise deletion was utilized within the regression analyses. Postestimation statistics, including *R*^2^, AIC, and BIC, were used for model selection.

## Results

Table 4 displays the results of the multiple regression analysis predicting student's knowledge of the confidentiality of on-campus resources available within the University of California system. The final model with perceived training effectiveness displayed the lowest AIC and BIC of the three models, as well as the highest *R*-squared.

**Table 4.** Regression Analyses Predicting Knowledge of On-Campus Resource Confidentiality (*N* = 677).

	Unadjusted coefficients	Social identities (*SE*)	With campus (*SE*)	With perceived training effectiveness (*SE*)
Race/ethnicity (white reference group)				
Asian	−0.363 (0.129)***	−0.254 (0.136)*	−0.286 (0.141)**	−0.304 (0.139)**
Native American/Alaskan Native	−0.786 (0.443)*	−0.334 (0.451)	−0.473 (0.455)	−0.523 (0.451)
Black/African American	0.175 (0.305)	0.135 (0.298)	0.093 (0.299)	0.036 (0.295)
Hispanic/LatinX	−0.188 (0.146)	−0.119 (0.147)	−0.11 (0.151)	−0.156 (0.15)
Middle Eastern/North African	−0.368 (0.372)	−0.362 (0.365)	−0.425 (0.365)	−0.427 (0.359)
Other	−0.509 (0.539)	−0.061 (0.535)	−0.161 (0.539)	−0.284 (0.531)
Multiracial	−0.203 (0.163)	−0.079 (0.161)	−0.096 (0.162)	−0.133 (0.16)
UC Campus (Berkeley reference group)				
Davis	−0.279 (0.2)		−0.372 (0.201)*	−0.365 (0.199)*
Hastings	−0.32 (0.193)*		−0.175 (0.193)	−0.194 (0.191)
Irvine	0.028 (0.179)		0.071 (0.178)	0.03 (0.176)
Los Angeles	−0.477 (0.22)**		−0.328 (0.232)	−0.415 (0.23)*
Merced	−0.314 (0.231)		−0.369 (0.23)	−0.388 (0.227)*
Riverside	−0.808 (0.259)***		−0.774 (0.258)***	−0.747 (0.256)***
San Diego	0.278 (0.549)		0.188 (0.544)	0.164 (0.536)
San Francisco	−0.191 (0.231)		−0.136 (0.232)	−0.174 (0.23)
Santa Barbara	−0.284 (0.195)		−0.204 (0.196)	−0.261 (0.193)
Santa Cruz	−0.056 (0.764)		−0.318 (0.751)	−0.417 (0.74)
LGBQ (straight reference group)	0.205 (0.102)**	0.15 (0.103)	0.132 (0.104)	0.13 (0.102)
Men (women reference group)	−0.424 (0.113)****	−0.37 (0.115)***	−0.303 (0.117)***	−0.299 (0.115)*
Transfer students (nontransfer student reference group)	−0.581 (0.142)****	−0.522 (0.147)****	−0.522 (0.148)****	−0.45 (0.146)**
International students (domestic student references group)	−0.285 (0.149)*	−0.194 (0.162)	−0.15 (0.163)	−0.16 (0.161)
Graduate students (undergrad reference group)	0.224 (0.1)**	0.172 (0.107)	0.243 (0.119)**	0.21 (0.117)*
Perceived training Effectiveness (“very effective” reference group)				
Somewhat effective	0.124 (0.16)			0.026 (0.16)
Neutral	−0.391 (0.176)**			−0.367 (0.174)**
Not very effective	−0.432 (0.188)**			−0.473 (0.187)**
Not at all effective	−0.634 (0.259)**			−0.767 (0.258)***
Intercept		7.027 (0.194)****	7.098 (0.234)****	7.34 (0.271)****
*R* ^2^		0.0647	0.089	0.1243

* *p* < .1. ** *p* < .05. *** *p* < .01. **** *p* < .001.

A total of 667 observations were included in the analytical sample. Results from the final model demonstrated that knowledge of resource confidentiality is significantly associated with several social identities and other variables. These include certain racial groups, campus locations, gender identity, transfer student status, graduate student status, and perceived training effectiveness. Compared to White students, Asian students scored lower in the Title IX knowledge measurement (Coef.  = −0.304) while controlling for other social identities. Native American/Alaskan Native students also had a lower average score compared to White students in their Title IX knowledge, yet the racial effect was diminished when covariates were added into the model. In terms of UC campuses, UC Davis, UC Los Angeles, UC Merced and UC Riverside have a lower score than UC Berkeley. Among these campuses, students from UC Riverside had the lowest Title IX knowledge score on average (Coef.  = −0.747). Other campuses, including UC Hastings, UC Irvine, UC San Diego, UC San Francisco, UC Santa Barbara, and UC Santa Cruz, were not statistically different from UC Berkeley in terms of Title IX knowledge among students when adjusting for other covariates.

As for other social identities, LGBQ + students tend to have a higher score of Title IX knowledge, though this effect did not remain significant after adjusting for other covariates. Gender was another variable that consistently impacted the Title IX knowledge scores as male students on average scored nearly 0.3 points lower than female students. Regarding student status, transfer students have less Title IX knowledge compared to nontransfer students (Coef.  = −0.45), while graduate students scored 0.21 points higher than undergraduate students, controlling for other covariates. International students scored lower than domestic students in the bivariate analysis, yet the effect became insignificant after adjusting for other covariates.

Lastly, compared to students who thought the Title IX training was very effective, students who thought Title IX training was not as effective scored lower in Title IX knowledge. In fact, the less effective the students think Title IX training was, the fewer points they scored on the Title IX knowledge measurement. For instance, students who think Title IX training was “not at all effective” scored −0.767 points less on average than students who think Title IX training was “very effective;” this difference is 0.4 points larger than comparing students who held a neutral opinion toward the training with the reference group.

## Discussion

Student knowledge of confidential and nonconfidential resources for survivors of SVSH across UCs varies by social identity. On average, men, transfer students, and international students are less knowledgeable of which resources across UC campuses are confidential compared to women, nontransfer students, and domestic students. However, graduate students are on average more knowledgeable of which resources across UC campuses are confidential compared to undergraduate students.

Results suggest that students are able to accurately recognize their knowledge of on-campus resources as a result of the Title IX training provided to them, as the less effective a student perceived the Title IX training, the fewer points they scored on the Title IX knowledge measurement. The differences in knowledge of Title IX by gender and sexuality may be explained by differences in experience of sexual violence between these groups; as noted in the literature review, this topic disproportionality impacts women and LGBTQ+ students (Eisenberg et al., 2021; Klein & Martin, 2021). Variables such as transfer student status, international student status, and graduate student status may be influenced by student age or experience with U.S. higher education or the available resources in UC campuses. For example, international students may not be as aware of confidentiality policies regarding SVSH within U.S. universities as they are less likely to hear about such policies throughout their lifetime in a different country. Graduate students may have experienced similar or the same confidentiality policies during their undergraduate education, whereas transfer students often transfer from a community college and are students within the UC system for a shorter period of time compared to their nontransfer counterparts.

Based on such findings, transfer students, international students, and undergraduate students should be provided with more targeted training or education regarding the availability of resources on their individual campuses to reduce the knowledge gap among student groups. Reiterating this information through either required or optional trainings or orientation to student life will reduce the likelihood that students of these groups will not know where to seek resources in the case of SVSH directed toward themselves or their peers.

Student awareness of confidential and nonconfidential resources for survivors of sexual violence, particularly before they experience such incidents, plays a pivotal role in averting situations of institutional betrayal. Without understanding which campus resources are required to report experiences to the Title IX office, or that seeking assistance from the Title IX office may open an investigation, students who do not wish to proceed with such processes may inadvertently open themselves up to a potentially traumatic experience. As students at several campuses across the UC system (UC Davis, UCLA, UC Merced, and UC Riverside) were less likely to correctly identify confidential on-campus resources, the examination of training policies regarding campus resources within these specific campuses would be useful in understanding why such differences are present in the data. By increasing student knowledge of resources available to survivors of sexual violence, experiences of institutional betrayal may be systematically prevented, as students who later experience sexual violence are able to choose to utilize resources that best fit their needs. In addition to increasing student knowledge of resources for survivors of sexual violence, institutions of higher education should decrease the challenges that may be associated with reporting an incident to the Title IX office and provide more resources for survivors that center on healing rather than retribution. Under Title IX, universities have a duty to protect students from experiences of sexual violence within their campuses, which should include more comprehensive and tailored services for survivors.

Finally, more research is needed to examine the effects and potential prevention of experiences of institutional betrayal after an experience of sexual violence within university campuses. For example, research regarding differential experiences with the Title IX office based on demographic characteristics may assist in the development of programs and policies that protect students who may be more or less likely to experience sexual violence during their time as a student.

### Limitations

It is important to note the limitations of the study. First, the study relied on self-reported SVSH or training-related variables such as the effectiveness of Title IX training, which may not be entirely accurate due to response biases. Furthermore, the instrument used to measure Title IX knowledge was not validated, which could have introduced bias and not adequately captured the concept of Title IX knowledge. For this reason, it is recommended that future research employ more sophisticated and validated instruments to measure this concept. The dataset utilized for the study is also not representative of the full UC student population. For example, survey respondents were more likely to be LGBQ or women compared to the overall UC student population, and the study sample is not perfectly representative of the UC student population by race and ethnicity (Fall Enrollment at a Glance, 2023). Compared to the total number of students at the school, UC Irvine, UC Merced, and UC San Francisco were overrepresented within the dataset, whereas UC Los Angeles, UC San Diego, and UC Santa Cruz were underrepresented in the dataset (Fall Enrollment at a Glance, 2023). Survey weights were not used within the analysis, and future research should examine the sample population compared to the full UC population. Finally, the study did not account for certain variables that could have impacted the outcome. For instance, the study did not distinguish between in-person and online training sessions, which could affect the content and effectiveness of the training. Additionally, the study did not consider the timing of participants’ experiences with Title IX training, particularly before or after the COVID-19 pandemic. The pandemic has significantly impacted access to resources, both on and off-campus, which could have influenced participants’ perceptions and experiences with Title IX training.

## Conclusion

Differences in knowledge of confidential and nonconfidential resources for survivors of SVSH across University of California campuses have the potential to impact survivors’ experiences, including the possibility of institutional betrayal. Variations in student knowledge suggest that nontransfer students, graduate students, students within specific UC campuses, and students who are women are more prepared to access desired services in the case of SVSH. Students tend to accurately identify the quality of the required Title IX training in its ability to educate them about on-campus resources. Additional research is needed to understand the intricacies of the impact that the required Title IX training has on student survivors across the UC system.
